# Learning and reasoning with graph data

**DOI:** 10.3389/frai.2023.1124718

**Published:** 2023-08-22

**Authors:** Manfred Jaeger

**Affiliations:** Department of Computer Science, Aalborg University, Aalborg, Denmark

**Keywords:** graph data, representation learning, statistical relational learning, graph neural networks, neuro-symbolic integration, inductive logic programming

## Abstract

Reasoning about graphs, and learning from graph data is a field of artificial intelligence that has recently received much attention in the machine learning areas of graph representation learning and graph neural networks. Graphs are also the underlying structures of interest in a wide range of more traditional fields ranging from logic-oriented knowledge representation and reasoning to graph kernels and statistical relational learning. In this review we outline a broad map and inventory of the field of learning and reasoning with graphs that spans the spectrum from reasoning in the form of logical deduction to learning node embeddings. To obtain a unified perspective on such a diverse landscape we introduce a simple and general semantic concept of a model that covers logic knowledge bases, graph neural networks, kernel support vector machines, and many other types of frameworks. Still at a high semantic level, we survey common strategies for model specification using probabilistic factorization and standard feature construction techniques. Based on this semantic foundation we introduce a taxonomy of reasoning tasks that casts problems ranging from transductive link prediction to asymptotic analysis of random graph models as queries of different complexities for a given model. Similarly, we express learning in different frameworks and settings in terms of a common statistical maximum likelihood principle. Overall, this review aims to provide a coherent conceptual framework that provides a basis for further theoretical analyses of respective strengths and limitations of different approaches to handling graph data, and that facilitates combination and integration of different modeling paradigms.

## 1. Introduction

Graphs are a very general mathematical abstraction for real world networks such as social-, sensor-, biological- or traffic-networks. These types of networks often generate large quantities of observational data, and using machine learning techniques to build predictive models for them is an area of substantial current interest. Graphs also arise as abstract models for knowledge, e.g., in the form of semantic models for a logic knowledge base, or directly as a knowledge graph. In most cases, an appropriate representation as a graph will require that one goes beyond the fundamental graph model, and allows that nodes are annotated with attributes, and that there are several distinct edge relation.

Evidently, in machine learning the main interest is in learning from graph data, whereas in knowledge representation and reasoning the primary focus is on deductive reasoning. However, both learning and reasoning play a role in all disciplines: making class label predictions from a learned model is a highly specialized (and limited) form of reasoning, and learning logic rules from examples has a long history in symbolic AI. It is the goal of this review to survey the large and diverse area of approaches for learning and reasoning with graphs in different areas of AI and adjacent fields of mathematics. Given the scope of the subject, our discussion will be mostly at a high, conceptual level. For more technical details, and more comprehensive literature reviews, we will point to relevant specialized surveys. The main objective of this review is to establish a coherent formal framework that facilitates a unified analysis of a wide variety of learning and reasoning frameworks. Even though this review aims to cover a broad range of methods and disciplines, there will be a certain focus on graph neural networks (GNNs) and statistical relational learning (SRL), whereas the fields of graph kernels and purely logic-based approaches receive a little less attention than they deserve.

The following examples illustrate the range of modeling, learning, and reasoning approaches that we aim to cover in this review. Each example describes a general task, a concrete instance of that task, and a particular approach for solving the task. The examples should not be construed in the way that the described solution approach is the only or even most suited one to deal with the given task. The intention is to illustrate the diversity of tasks and solution techniques.

Example 1.1. (Node classification with graph neural networks) One of the most common tasks in machine learning with graphs is *node classification*, i.e., predicting an unobserved node label. A standard instance of such a classification task is subject prediction in a bibliographic data graph: nodes are scientific papers that are connected by citation links, and the node labels consist of a subject area classification of the paper. In the *inductive* version of this task, one is given one or several training graphs containing labeled *training nodes*. The task is to learn a model that allows one to predict class labels of unlabeled nodes that are not already contained in the training graphs. For example, the training graph may consist of the current version of a bibliographic database, whereas the unlabeled nodes are new publications when they are added to the database. In the *transductive* version of the task, both the labeled training nodes and the unlabeled test nodes reside in the same graph, which is already fully known at the time of learning. This is the case when an incomplete subject area labeling is to be completed for a given bibliographic database. Graph neural networks are a state-of-the-art approach to solve such classification tasks (e.g., Niepert et al., 2016; Hamilton et al., 2017; Welling and Kipf, 2017; Veličković et al., 2018).

Example 1.2. (Link prediction via node embeddings) Here the task is to predict whether two nodes are connected by an edge. This prediction problem is usually considered in a transductive setting, where all the nodes and some of the edges are given, and edges between certain test pairs of nodes have to be predicted. Bibliographic data graphs are again a popular testbed for link prediction approaches (Kipf and Welling, 2016; Pan et al., 2021). *Recommender systems* also can be seen as handling a link prediction problem: the underlying graph here contains *user* and *product* nodes, and edges connect users with products that the user has bought (or provided some other type of positive feedback for). The link prediction problem then amounts to predicting positive user/product relationships that have not yet been observed. Numerous different link prediction approaches exist (Kumar et al., 2020 gives a comprehensive survey). A variety of different approaches is based on constructing for each node in the graph a *d*-dimensional real-valued *embedding vector*, and to score the likelihood of the existence of an edge between two nodes by considering the proximity (according to a suitable metric) of their embedding vectors. This general paradigm encompasses approaches such as matrix factorization (Koren and Bell, 2015) and random walk based approaches (Perozzi et al., 2014; Grover and Leskovec, 2016).

A good and concise monograph that covers the modern machine learning methods described in the preceding two examples is (Hamilton, 2020).

Example 1.3. (Graph Classification with inductive logic programming) One may also want to predict a class label associated with a whole graph. A classic example is predicting properties of molecules, where molecules are represented as graphs consisting of nodes representing the atoms, and links representing bonds between the atoms. The famous *Mutagenesis* dataset (Srinivasan et al., 1996), for example, consists of 188 molecules with a Boolean *mutagenic* class label. A predictor for this label may be given in the form of a logic program, such as the following:


$$\begin{array}{ccc} carbon\_path(A,B) & \leftarrow & carbon(A),carbon(B),bond(A,B)\\ carbon\_path(A,B) & \leftarrow & carbon(B),carbon\_path(A,C),bond(B,C)\\ carbon\_cycle & \leftarrow & carbon\_path(A,A)\\ mutagenic & \leftarrow & carbon\_cycle \end{array}$$

(this program here is purely given for expository purposes, and does not resemble realistic classification programs for this task). A particular molecule specified by a list of *ground facts*, such as *carbon*(*at*_1), *carbon*(*at*_2), *nitrogen*(*at*_3), …, *bond*(*at*_1, *at*_3), would then be classified as mutagenic, if *mutagenic* can be proven by the program from the ground facts. This will be the case if and only if the molecule contains a cycle consisting of carbon atoms. Parts of the program (e.g., the definitions of carbon path and cycle) may be provided by experts as background knowledge, whereas other parts (e.g., the dependence of mutagenic on the existence of a carbon cycle) would be learned from labeled examples.

Example 1.4. (Graph similarity and classification with graph kernels) The problem of graph classification (especially in bio-molecular application domains) has also extensively been approached with kernel techniques. Graph kernels (see Kriege et al., 2020 for an excellent survey) are functions *k* that map pairs of graphs *G, H* to a value *k*(*G, H*)∈ℝ that is usually interpreted as a similarity measure for *G* and *H*, and which must be of the form *k*(*G, H*) = ϕ(*G*)·ϕ(*H*) for some finite or infinite dimensional real-valued feature vectors ϕ(*G*), ϕ(*H*). The graph kernel can then be used for graph classification by using it as input for a support vector machine classifier. Based on its interpretation as a similarity measure, graph kernels can also support other types of similarity-based analyses, e.g., clustering. Most graph kernels are defined by an explicit definition of the mapping from graphs *G* to feature vectors ϕ(*G*). Important examples are the *Weisfeiler-Lehman kernel (WLK)* (Shervashidze et al., 2011), the *graphlet kernel* (Shervashidze et al., 2009), and the *random walk kernel* (Gärtner et al., 2003). The components of the feature vectors in the first two contain statistics on the occurrence of local neighborhood structures in *G*, whereas the features of the random walk kernel represent statistics on node label sequences that are generated by random walks on the graph.

The previous examples were concerned with specific prediction tasks. In the following examples we move toward more general forms of reasoning.

Example 1.5. (Probabilistic inference with SRL) The task is to learn a probabilistic graph model that supports a rich class of queries. An example is a probabilistic model for the genotypes of people in a *pedigree* graph. The model should support a spectrum of queries. A basic type are conditional probability queries of the form: given (partial) genotype information for some individuals, what are the probabilities for the genotypes of the other individuals? This is still very similar to the node classification task of Example 1.1. A query that goes beyond what has been described in previous examples is a *most probable explanation (MPE)* query: again, given partial genotype information, what is the most probable joint configuration of the genotypes for all individuals? *Statistical relational learning (SRL)* approaches such as *Relational Bayesian Networks (RBNs)* (Jaeger, 1997), *Markov logic networks (MLNs)* (Richardson and Domingos, 2006), or *ProbLog* (De Raedt et al., 2007) provide modeling and inference frameworks for solving such tasks.

Example 1.6. (Logical reasoning with first-order logic) Going beyond the flexible, but still rather structured type of queries considered in Example 3.6, we can consider more general logical reasoning tasks. There are no standard example instances of this, so we illustrate this task and its solution by deduction in first-order logic by the following example: given the following knowledge about a social network:

- Every user follows at least one other user. Expressed as a first-order logic formula, this reads as:

(1) $$\begin{array}{l} \forall x\exists yfollows(x,y). \end{array}$$

- There is a user who is not followed by anyone:

(2) $$\begin{array}{l} \exists y\neg \exists x\text{follows}\left(x,y\right) \end{array}$$

Does this knowledge imply that there must be a user who has at least two followers, or there must be at least four different users:


(3) $$\begin{array}{l} (\exists y\exists^{\geq 2}xfollows(x,y))\vee \exists^{\geq 4}x? \end{array}$$

Considering first all finite graphs, one finds that when (1) and (2) are true there must be a node with at least two incoming edges, i.e., the first part of the disjunction in (3) is true. When the graph is infinite, then this implication no longer holds, but then the second disjunct of (3) will be true (where the number 4 may be replaced with any natural number). Thus, we find that (1) and (2) logically entail (3). This inference can be performed by automated theorem provers. For example the SPASS prover (Weidenbach et al., 2009) can answer our query.

Example 1.7. (Limit behavior of random graphs) Many probabilistic models have been developed for the temporal evolution of growing networks. The simplest possible model is to assume that every new node is connected to the already existing nodes with a fixed probability *p*, and these connections are formed independently of each other. One may then ask how the probabilities of certain graph properties develop, as the size of the graph grows to infinity. Classic results of the Erdős-Rényi random graph theory establish, for example, that the limiting probability for the evolving graph to be connected is 1 (Erdős and Rényi, 1960) [the actual results of the Erdős-Rényi random graph theory are actually much more sophisticated, as they pertain to models where the edge probability is a function *p*(*n*) of the number *n* of vertices]. Similarly, it is known that the probability of every property that can be expressed in first-order logic converges to either 0 or 1 (Fagin, 1976). Reasoning about such limiting probabilities goes beyond reasoning about all graphs as in Example 1.6, since we now consider probability distributions over infinite sequences of graphs. Some types of queries about the limit behavior of random graphs are formally decidable. This is the case, for example, for the limit probability of a first-order sentence (Grandjean, 1983). However, the computational complexity of these reasoning tasks puts them outside the range of practically feasible implementations.

**Figure 1:**
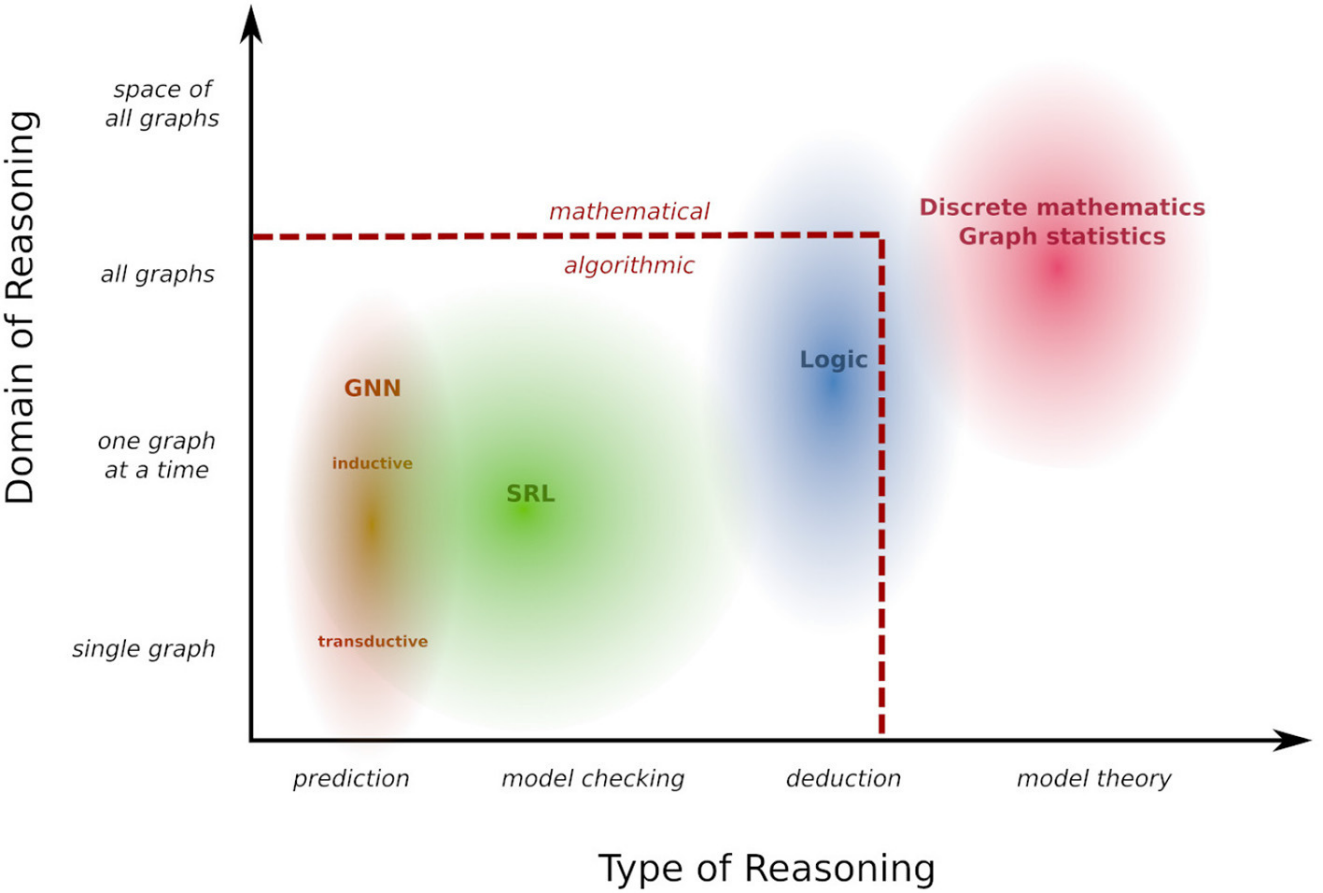
Reasoning landscape.

Figure 1 arranges the landscape of reasoning scenarios we have considered in the preceding examples in two dimensions: one dimension characterizes the domain of graphs that we reason about: at the bottom of this dimension is the transductive setting of Examples 1.1 and 1.2 in which reasoning is limited to a single given graph. At the next level, labeled “one graph at a time,” any single reasoning task is about a specific graph, but this graph under consideration can vary without the need to change or retrain the model. This corresponds to the inductive setting in Example 1.1, as well as the tasks described in Examples 1.4 and 3.6. At the third level, the reasoning concerns several or all graphs at once (Example 1.6). Finally, we may go beyond reasoning about properties of individual graphs, and consider global properties of the space of all graphs. This is exemplified by Example 1.7, where the reasoning pertains to the relationship between different probability distributions on graphs. These informal distinctions about the domain of reasoning will be partly formalized by technical definitions in Section 5.

The second dimension in Figure 1 is correlated with the first, but describes the type of reasoning that is performed. The most restricted type here is to perform a narrowly defined prediction task such as node classification or link prediction. Based on the distinction between “model checking” and “theorem proving” promoted by Halpern and Vardi (1991) we label the next level as “model checking”: this refers to evaluating properties of a single given structure, where the class of properties that can be queried is defined by a rich and flexible query language. “Deduction” then refers to (logical) inference about a class of structures, and in “model theory” such a class of structure becomes the object of reasoning itself. Within this two-dimensional schema, Figure 1 indicates what areas of reasoning are covered by different types of frameworks. The reasoning landscape here delineated extends beyond the boundaries of what can currently be tackled with automated, algorithmic reasoning methods in AI, and extends to human mathematical inference. In the remainder of this review we will focus on algorithmic reasoning. However, it is an always open challenge to extend the scope of what can be accomplished by algorithmic means.

Figure 1 focuses on reasoning rather than learning. This is for two reasons: first, reasoning covers a somewhat wider ground that includes scenarios (e.g., logical deduction) not yet supported by learning methods. Second, a taxonomy of learning scenarios in terms of data requirements and learning objectives will naturally follow from the reasoning taxonomy (cf. Section 6). The link between reasoning and learning is a *model* that can be learned from data and that supports the reasoning tasks under consideration. In this review we use a general probabilistic concept of a model formally defined in in Section 3, that allows us to express a wide range of reasoning tasks as different forms of querying a model (Section 5). Similarly, a wide range of learning scenarios can be understood in a coherent manner as constructing models from data under a maximum likelihood objective (Section 6). This review is partly based on Jaeger (2022), which already contains an in-depth exploration of GNN and SRL methods for learning and reasoning with graphs. The current review takes a much broader high-level perspective. It contains fewer technical details on GNNs and SRL, and instead develops a general conceptual framework for describing a much wider range of learning and reasoning frameworks.

## 2. Graphs

In this section we establish the basic definitions and terminology we use for graphs. Table 1 collects the main notations. Our graphs will actually be multi-relational, attributed hyper-graphs, but we just say shortly “graphs.”

**Table 1:** Overview of notation.

**[*n*]**	**The (node) set {1, …, *n*}**
R	Signature of relation symbols
R_*in*_, R_*out*_	Sets of designated input and output relations
*e, r*, …	(Lower case) specific symbols in R
*i, j*, …	Nodes
**i**, **j**, …	Tuples of nodes
*E, R*, …	(Upper case) interpretations of *e, r*, … in a specific graph
**R**	Tuple of interpretations for all *r*∈R
$\tilde{R},\tilde{R},$	Partial interpretation(s)
$\tilde{R},\tilde{R},$	$\tilde{R^{\prime }}$ Extends partial interpretation $\tilde{R}$
IntV(ℛ)	Set of interpretations of R over domain *V*
G(V,R)	Set of graphs for signature R with domain *V*
G(<∞,R)	Set of all finite graphs for signature R
G(R)	Set of all graphs for signature R
$\tilde{G}\left(\ldots \right)$	Corresponding sets of partial graphs
ΔG(V,R)	Space of probability distributions on G(V,R)
IntV(ℛ)	Set of completions of partial interpretation R
{|…|}	Delimiters for multisets

Different attributes and (hyper-) edge relations are collected in a *signature*: a set $R=\left\{r_{1},\ldots ,r_{m}\right\}$ of *relation symbols*. Each relation symbol has an *arity*(*r*_*i*_)∈{0, 1, …}. Relations of arity 0 are global graph attributes, such as *toxic* for a graph representing a chemical molecule. Relations of arity 1 are node attributes. Relations of arities ≥3, can in principle be reduced to a set of binary relations by materializing tuples as nodes. For example, the 3-ary relation *shortest_path*(*a, b, c*) representing that node *b* lies on the shortest path from *a* to *c* can be encoded by creating a new shortest path node *sp*, and three binary relations *start, on, end*, so that *start*(*sp, a*), *on*(*sp, b*), *end*(*sp, c*) are true. However, this leads to very un-natural encodings, and therefore we allow relations of arities ≥3.

A R-*graph* is a structure (*V*, **R**), where *V* is a finite or countably infinite set of nodes (also referred to as a *domain*), and **R** = (*R*_1_, …, *R*_*m*_) are the *interpretations* of the relation symbols: $R_{i}:V^{\text{arity}\left(r_{i}\right)}\to \left\{0,1\right\}$. In the case of *arity*(*r*_*i*_) = 0 this is just a constant 0 or 1.

We write *IntV*(*r*) for the set of possible interpretations of the relation symbol r∈R over the domain *V*, and $\text{Int}V\left(R\right)=\times_{r\in R}\text{Int}V\left(r\right)$ for the set of all interpretations of the whole signature R. In most cases it is sufficient to consider the domains *V* = [*n*]: = {1, …, *n*} for *n*∈ℕ. We use *i, j*, … as generic symbols for nodes, and bold face **i**, **j**, … for tuples of nodes. We here use somewhat logic-inspired terminology and notation. Taking the logic conventions even further, we can equivalently define an interpretation of *r* as an assignment of true/false values to *ground atoms* *r*(**i**) [**i**∈|*V*|^*arity*(*r*)^]. Going in the opposite direction toward the terminological conventions of neural networks, such an interpretation also can be seen as a |*V*| × ⋯ × |*V*|-dimensional (*arity*(*r*) many factors) tensor.

In the following, we usually take the signature R as given by the context, and do not refer to it explicitly, thus saying “graph” rather than R-*graph*, and also abbreviating IntV(R) by *IntV* Also, when the intended meaning is clear from the context, we use the simple term “relation” to either refer to a relation symbol *r*, or an interpretation *R* in a specific graph.

Note that according to our definitions all relations are *directed*. Undirected edges/relations are obtained as the special case where the interpretation *R*_*i*_ for a tuple **i** only depends on the elements of **i**, not their order. Furthermore, only Boolean node attributes are permitted. Multi-valued attributes can be represented by multiple Boolean ones in a one-hot encoding.

For a given domain *V* and signature R we denote with G(V,R) the set of all R-graphs with domain *V*, with G(<∞,R) the set of all finite R-graphs, and with G(R) the set of all graphs, also allowing (countably) infinite domains *V*. As a basis for probabilistic graph models, we denote with ΔG(V,R) the set of probability distributions over G(V,R) (in the case of infinite *V* this must be based on suitable measure-theoretic definitions that we do not elaborate here).

## 3. Models

We introduce a general concept of a model, so that all reasoning tasks become different forms of querying a model. Ours will be a high-level, purely semantic notion of a model that imposes no restrictions on how models are represented, implemented or constructed. Our model definition is probabilistic in nature. As we shall see, this still allows us to capture purely qualitative, logic-based frameworks, although at the cost of casting them in a slightly contrived way into the probabilistic mold via extreme 0, 1-valued probabilities. Roughly speaking, a model in our sense will be a mapping from partly specified graphs to probability distributions over their possible completions.

A *partial graph* is a structure $\left(V,\tilde{R}\right)$ where *V* is a finite or countably infinite domain, and $\tilde{R}=\left({\tilde{R}}_{1},\ldots ,{\tilde{R}}_{m}\right)$ are *partial interpretations* of the relation symbols: ${\tilde{R}}_{i}:V^{\text{arity}\left(r_{i}\right)}\to \left\{0,1,?\right\}$. We write ${\tilde{R}}_{i}≼{\tilde{R}}_{i}^{\prime }$ (${\tilde{R}}_{i}^{\prime }{\tilde{R}}_{i}$ *extends* ${\tilde{R}}_{i}$) nif


$$\forall i:{\tilde{R}}_{i}(i)\neq ?\;\Rightarrow \;{{\tilde{R}}^{\prime }}_{i}(i)={\tilde{R}}_{i}(i).$$

If ${\tilde{R}}_{i}≼{\tilde{R}}_{i}^{\prime }$ for *i* = 1, …, *m* we write ${\tilde{R}}_{i}≼{\tilde{R}}_{i}^{\prime }$.

$\tilde{G}\left(V,R\right)$ denotes the set of all partial R-graphs with domain *V*, and $\tilde{G}\left(<\infty ,R\right)$ the set of all finite partial R-graphs. Finally, $\text{Int}\left(V,\tilde{R}\right)\left(R\right)$ denotes the set of complete interpretations $R\in Int_{V}ℛ$ with ${\tilde{R}}_{i}≼R$.

Definition 3.1. A R-model M=(I,μ) consists of

- a subset $I\subseteq \tilde{G}\left(<\infty ,R\right)$
- a mapping

$$\mu :\;(V,\tilde{R})\mapsto P_{\left(V,\tilde{R}\right)}\in \Delta G(V,ℛ)$$

defined for all $(V,\tilde{R})\in ℐ$, such that


$$P_{\left(V,\tilde{R}\right)}(Int_{\left(V,\tilde{R}\right)}\left(ℛ)\right)=1.$$

In the case where the input just consists of a domain *V* (i.e., $\tilde{ℛ}$ is completely unspecified), we simply write *P*_*V*_ for $P_{\left(V,\tilde{R}\right)}$.

Definition 3.1 has several important special cases: when a model is for classification of a specific label relation *l*, then I=G(<∞,ℛ∖{l}).. The model then defines for every input graph with complete specifications of relations r∈R\{l} a distribution over interpretations *L* for *l*. We refer to such models as *discriminative*. A model that, in contrast, takes as input only a finite domain, i.e., I=G(<∞,∅), and thereby maps a domain *V* to a probability distribution over G(V,R) is called *fully generative*. In between these two extremes are models where R is partitioned into a set of *input* relations R_*in*_ and output relations R_*out*_, and $I=G\left(<\infty ,R_{\text{in}}\right)$. This is the typical case for SRL models. We refer to such models as *conditionally generative* (discriminative then is just a borderline case of conditionally generative). In all these special cases the input graph contains complete specifications of a selected set of relations. This covers many, but not all models used in practice: e.g., models for link prediction (cf. Example 3.3 below) operate on inputs with partial specifications of the edge relation. Transductive models are characterized as the special case |I| = 1.

**Figure 2:**
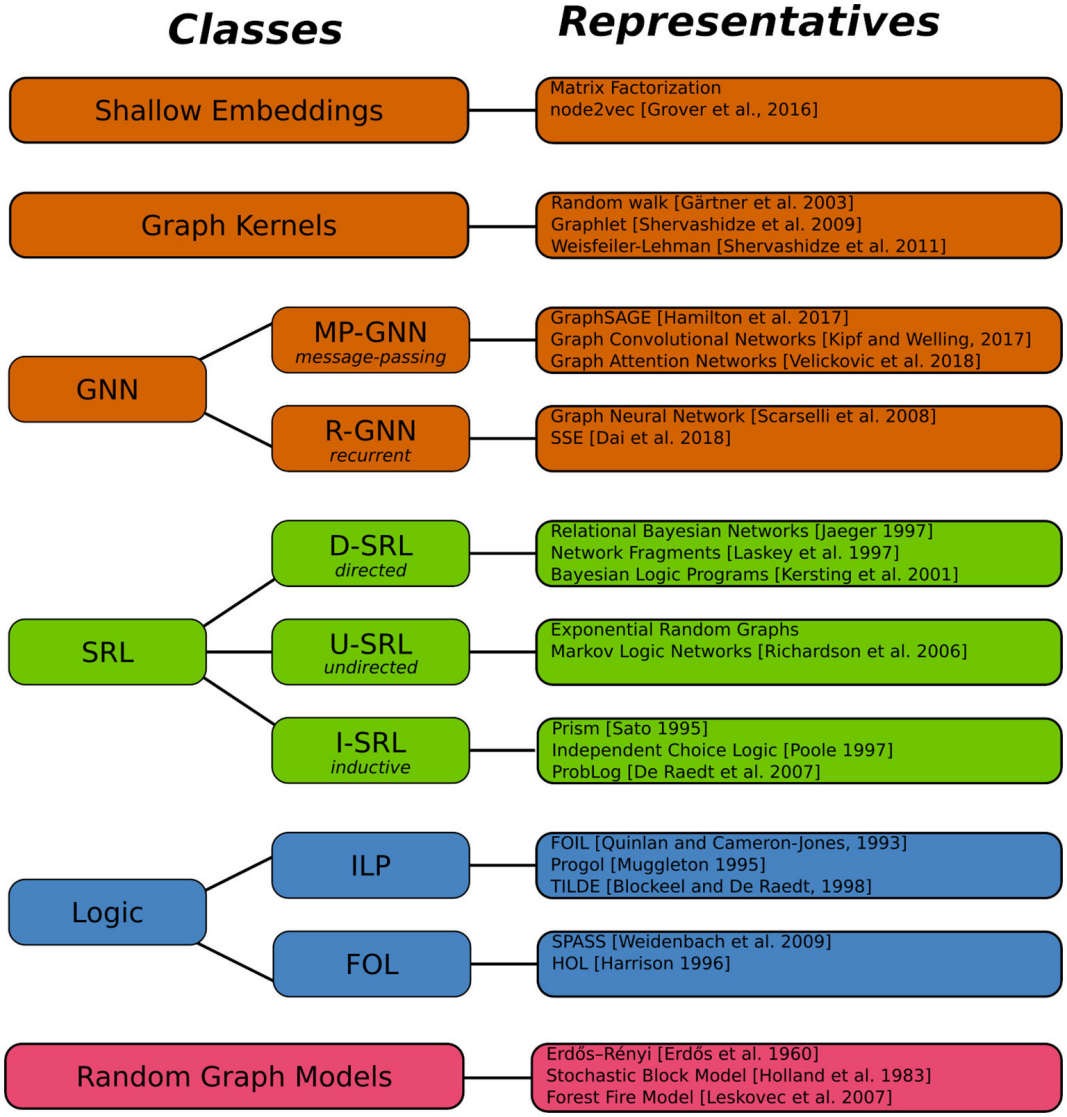
Framework classes and representatives.

The abstract definition 3.1 accommodates a multitude of concrete approaches for learning and reasoning about graphs. We call a *modeling framework* any approach that provides computational tools for representing, learning, and reasoning with models. Figure 2 gives an overview of several classes of modeling frameworks, and representatives for each class. The selection of representatives does not attempt a complete coverage of even the most important examples. In the following we continue the examples of Section 1 to illustrate how these frameworks indeed fit into our general Definition 3.1.

Example 3.2. (Node classification; GNNs) In the standard node classification scenario, the signature R consists of one binary edge relation *e*, observed node attributes **a** = *a*_1_, …, *a*_*l*_, and a node label *l*. A partial input graph has the form $\left(V,\tilde{R}\right)$, where in the partial interpretation $\tilde{R}=\left(E,A,\tilde{L}\right)$ the edge relation and node attributes are fully observed, and the node label is unobserved for some (possibly all) nodes. Given a Graph neural networks then define label probabilities


$$P(l(i)|(V,(E,A)))\;(i:\tilde{L}(i)=?).$$

for the unlabeled nodes. Since these predictions are independent for all nodes, they define a distribution over completions *L* of $\tilde{L}$ via


(4) $$P_{\left(V,\tilde{ℛ}\right)}(L)=\Pi_{i:\tilde{L}(i)=?}P(l(i)=L(i)|(V,(E,A))).$$

In Figure 2, the class of GNN frameworks is divided into the sub-classes of *message-passing* and *recurrent* GNNs. MP-GNNs compute node feature vectors in a fixed number of iterations, whereas R-GNNs perform feature updates until a fixed point is reached. We return to this distinction in Section 4.2.

Example 3.3. (Link prediction; shallow embeddings) In its most basic form, a transductive link prediction problem is given by a single input graph I={(V,Ẽ)} with an partially observed edge relation. Usually, all edges that are not observed as existent are candidates for being predicted, so that Ẽ(*i, j*)∈{1, ?} for all *i, j*∈*V* (in practice, however, represented in the form Ẽ(*i, j*)∈{1, 0}, with the understanding that Ẽ(*i, j*) = 0 still allows the prediction *E*(*i, j*) = 1). *Shallow embeddings* construct for each node *i*∈*V* an embedding vector *em*(*i*), and define a scoring function *score*[*em*(*i*), *em*(*j*)] for the likelihood of *E*(*i, j*) = 1. As the objective usually is to just rank candidates edges, this score need not necessarily be probabilistic [concrete examples are the dot product or cosine similarity of *em*(*i*), *em*(*j*)]. However, turning the scores into probabilities by applying a sigmoid function does not affect the ranking and hence we may assume that the link prediction model defines edge probabilities *P*[*e*(*i, j*)|(*V*, Ẽ)] (*i, j*:Ẽ(*i, j*) = ?). Via the same implicit independence assumptions as in the previous example, then a distribution in the sense of Definition 3.1 is defined as


(5) $$P_{\left(V,\tilde{E}\right)}(E)=\Pi_{i,j:\tilde{E}(i,j)=?}P(e(i,j)=E(i,j)|(V,\tilde{E})).$$

Example 3.4. (ILP) A logic program as shown in Example 1.3 can be interpreted as a model in our sense. For any input $\left(V,\tilde{R}\right)$, where $\tilde{R}$ is specified by a list of true ground facts, the program defines the unique interpretation R^*^ in which a ground fact *r*(**i**) is true, iff it is provable from the program and $\tilde{R}$. This can be expressed in the form of a degenerate probability distribution with


$$P_{\left(V,\tilde{R}\right)}(R^{*})=1.$$

The set I of possible inputs for this model consists of all $\left(V,\tilde{R}\right)$ where $\tilde{R}$ is defined by positive ground facts only, i.e., $\tilde{R}\left(i\right)\in \left\{1,?\right\}$ for all $\tilde{R},i$.

Example 3.5. (Graph Classification; graph kernels) A graph kernel *k*(*G, H*) alone is not a model in our sense. However, a support-vector machine classifier built on the kernel (denoted *k*-SVM) is such a model: a *k*-SVM maps graphs to label values in {0, 1}, which similar to Example 3.4 can be seen as a degenerate probabilistic model. Alternatively, since the *k*-SVM actually produces numeric scores for the labels (as the distance to the decision boundary), one can transform the score by a sigmoid function to non-degenerate probability values.

Example 3.6. (SRL) To solve the probabilistic inference tasks of Example an SRL model will be defined for input structures (*V, E*) with a fully observed edge relation *e* defining the pedigree structure. The model will then define a distribution *P*_(*V, E*)_(**A**) over interpretations **A** of node attributes **a**. In a typical solution for this type of problem this will be done by defining a marginal distribution over genotypes for the individuals in *V* whose parents are not included in *V*, and a conditional distribution over child genotypes given parent genotypes. In order to solve the reasoning tasks described in Example, the framework must support queries about conditional probabilities of the form $P_{\left(V,E\right)}\left(a\left(i\right)|\tilde{A}\right)$ (genotype probabilities for individual *i* given partial information $\tilde{A}$ about genotypes in the pedigree) and $argmax_{A}P_{\left(V,E\right)}(A|\tilde{A})$ (MPE inference).

The model outlined here falls into the sub-class of *directed* SRL frameworks, where the distributions defined by a model can be represented in the form of directed probabilistic graphical models. Other important sub-classes of SRL distinguished in Figure 2 are frameworks based on *undirected* probabilistic graphical models, and probabilistic generalizations of inductive logic programming. Because of their close relationship with the most popular undirected SRL framework, Markov logic networks, Figure 2 also lists *exponential random graph models* under the U-SRL class, even though in terms of historical background and applications, exponential random graphs rather fall into the random graph model category.

Example 3.7. (First-order logic) A logical knowledge base *KB* as exemplified by (1) and (2) in Example 1.6 can be seen as a discriminative model in our sense: for any graph *G* = (*V*, **R**)∈G(R) the semantics of the logic defines whether *KB* is true in *G*.^1^ To formalize this as a discriminative model we augment the signature R with a binary graph label *l*_*KB*_, set I = G(R), and *P*_*G*_(*l*_*KB*_ = 1) = 1 if *KB* is true in *G*, and *P*_*G*_(*l*_*KB*_ = 0) = 1, otherwise. Logical inference as described in Example 1.6 then amounts to determining whether for all graphs *G* in which (3) is false one has *P*_*G*_(*l*_*KB*_ = 0) = 1. This is a probabilistic rendition of the task of automated theorem proving.

Example 3.8. (Random graph models) Classical random graph models are generative models in our sense for the signature R = {*e*} containing a single edge relation.

The preceding examples show that the quite simple Definition 3.1 is sufficient as a unifying semantic foundation for a large variety of frameworks for reasoning about graphs. Of course, most of these frameworks also (or primarily) are designed for learning models from graph data, but our initial focus here is on modeling and reasoning, before we turn to learning in Section 6.

## 4. Modeling tools

Our Definition 3.1 of a model is completely abstract and does not entail any assumptions or prescriptions about the syntactic form and computational tools for model specification and reasoning. In the following, we consider several key modeling elements that are used across multiple concrete frameworks.

### 4.1. Factorization

We first consider conditionally generative models that for an input graph (*V*, **R**_*in*_) define a probability distribution *P*_(*V*,_**R**__*in*_)_ (in the following abbreviated as *P*) over interpretations **R**_*out*_ of output relations. Enumerating R_*out*_ as *r*_1_, …, *r*_*n*_, one can factorize *P* as


(6) $$\begin{array}{l} P(R_{out}|R_{in})=P(R_{1}|R_{in})P(R_{2}|R_{1},R_{in})P(R_{3}|R_{1},R_{2},R_{in})\cdots \\ \;P(R_{n}|R_{1},\ldots ,R_{n-1},R_{in}). \end{array}$$

Thus, a generative model is decomposed into a product of discriminative models. This factorization at the relation level is often used in SRL models that are based on directed graphical models (Breese et al., 1994; Ngo and Haddawy, 1995; Jaeger, 1997; Laskey and Mahoney, 1997; Friedman et al., 1999; Kersting and De Raedt, 2001; Heckerman et al., 2007; Laskey, 2008).

Individual discriminative factors *P*(*R*_*k*_|*R*_1_, …, *R*_*k*−1_) can further be decomposed into a product of atom probabilities:


(7) $$\begin{array}{l} P(R_{k}|R_{1},\ldots ,R_{k-1},R_{in})=\prod_{i\in V^{arity(R_{k})}}P(r_{k}(i)=\\ \;R_{k}(i)|R_{1},\ldots ,R_{k-1},R_{in}) \end{array}$$

We have seen that this factorization is implicitly present in GNNs (4) and shallow embeddings (5). Unlike (6), which is a generally valid application of the chain rule, the factorization (7) represents a quite restrictive assumption that the *R*_*k*_-atoms are conditionally independent given relations *R*_1_, …, *R*_*k*−1_. This leads to some challenges, e.g., for modeling *homophily* properties of *R*_*k*_. A useful strategy to address such limitations of (7) is to include among the *R*_1_, …, *R*_*k*−1_ *latent relations* that only serve to induce dependencies among the *R*_*k*_-atoms.

Frameworks that are based on undirected probabilistic models, notably *exponential random graph models* and the closely related *Markov logic networks (MLNs)* (Richardson and Domingos, 2006), decompose the joint distribution *P*(**R**_*out*_) into factors that are defined by *features* *F*(**i**) of node tuples **i**. Examples of such features are the degree of a node *F*(*i*) = |{*j*|*edge*(*i, j*)}|, or, in MLNs, 0,1-valued featured expressed by Boolean formulas over ground atoms, e.g., *F*(*i, j*) = *edge*(*i, j*)∧*r*(*i*)∧¬*r*(*j*). Every such feature has an arity, and the distribution defined by features *F*_1_, …, *F*_*K*_ then is


(8) $$P(R_{out})=\frac{1}{Z}\text{exp}\left(\sum_{k=1}^{K}\sum_{i\in V^{arity(F)}}F_{k}(i)(R_{out},R_{in})\right),$$

where *Z* is a normalizing constant, and on the right-hand side we make explicit that the feature is a function of the interpretations **R**_*out*_ and **R**_*in*_.

### 4.2. Feature construction

Both the basic factors *P*(*R*_*k*_(**i**)|*R*_1_, …, *R*_*k*−1_, **R**_*in*_) in (7) and *F*(**i**)(**R**_*out*_, **R**_*in*_) in (8) are functions that take as input a graph (*V*, **R**′) containing interpretations **R**′ for some subset ${ℛ}^{\prime }\subseteq ℛ={ℛ}_{in}\cup^{\text{​}}{ℛ}_{out}$, and return a mapping of entity tuples **i** into ℝ. Such entity feature functions also lie at the core of many other modeling frameworks than those that use these features inside a probabilistic factorization approach: graph neural networks define a sequence of node feature vectors (a.k.a. embedding or representation vectors). Each component in such a vector is a feature function in our sense. A FOL formula with *k* free variables defines a 0,1-valued feature of arity *k*.

Graph kernels, by definition, are based on feature functions defined on whole graphs, i.e., features of arity zero in our sense. These features, however, often are aggregates of features defined at the single node level (e.g., in the Weisfeiler-Lehman kernel), or *k*-tuple level (e.g., in the graphlet kernel, whose features are closely related to the MLN features).

In many cases, feature functions are nested constructs where complex features are built from simpler ones. An important consideration for feature construction is whether they are used in models for transductive or inductive reasoning tasks. In the latter case the required generalization capabilities of the model imply that all features should be *invariant under isomorphisms*, i.e., $F\left(\tilde{i}\right)\left(Ṽ,{\tilde{R}}^{\prime }\right)=F\left(i\right)\left(V,R^{\prime }\right)$ whenever there is a graph isomorphism from (*V*, **R**′) to $\left(Ṽ,{\tilde{R}}^{\prime }\right)$ that maps **i** to $\tilde{i}$.

Table 2 summarizes some characteristics of the feature functions used in different frameworks. The column “Arity” indicates whether the framework uses features of graphs (0), nodes (1), or tuples of any arities ≥0. The remaining columns are addressed in the following sub-sections.

**Table 2:** Properties of feature functions underlying different frameworks.

**Framework**	**Arity**	**Initial**	**Aggregation**	**Final**
Shallow embedding	0,1	n/a	n/a	Shallow
Kernel	≥0	at	*div*	Deep, shallow
MP-GNN	0,1	id,at	Sum, mean, max,…	Deep
R-GNN	0,1	id,at	Sum, mean, max,…	Sat
D-SRL	≥0	at,rel	Noisy-or, mean,…	Deep
U-SRL	≥0	at,rel	n/a	Shallow
I-SRL	≥0	at,rel	∃	Sat
ILP	≥0	at,rel	∃	Sat
FOL	≥0	at,rel	∃, ∀	Deep
Random graph models	*div*	*div*	*div*	*div*

*div*: the framework class is too loosely defined to allow a meaningful entry.

#### 4.2.1. Initial features

**Figure 3:**
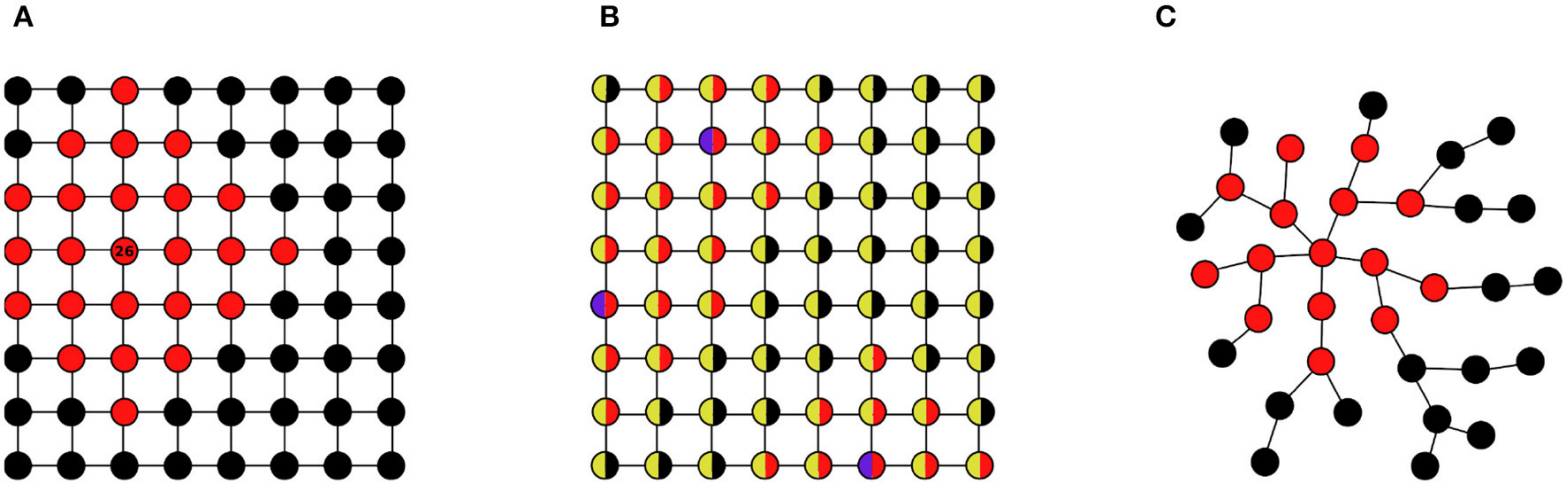
Initial node features and their use: **(A)** node identifiers; **(B)** node attributes; **(C)** vacuous.

All feature constructions start with some initial base features (we note that when here and in the following we talk about “construction,” then this is not meant to imply manual construction; it may very well be automated construction by a learner). Initial features often are node features. Here already important distinctions arise that essentially determine whether the model will have a transductive or inductive use. If one uses *unique node identifiers* as initial features (denoted “id” in the “Initial” column of Table 2), then models constructed from these features are not invariant under isomorphisms, and will be limited to transductive reasoning. As an example consider the feature *F*(*i*): “*i* is at most three edges away from node ‘26'.” This feature is illustrated by the red coloring of nodes in Figure 3A. While this feature can be useful for one specific graph (e.g., for predicting a node label), it does not generalize in a meaningful way to other graphs, even if they also contain a node with identifier “26.” Node identifiers are mostly used for transductive reasoning with GNNs. They are usually not used in SRL or kernel frameworks.

The most commonly used initial features are *node attributes* (“at” in Table 2). For example, if nodes have a color attribute with values “blue” and “yellow,” then with this initial features one can define a feature *F*(*i*): “*i* is at most two edges away from a blue node.” This feature, illustrated by a red coloring in Figure 3B, also applies to other graphs sharing the same signature R containing the blue/yellow attribute (we note that the regular grid structures of the graphs (a) and (b) are for illustrative clarity only; these features are in no way linked to such regular structures).

In some cases, there are no informative initial features used, or available. Formally, this can be expressed by a single *vacuous* node attribute that has a constant value for all nodes. Such a vacuous initial feature can still be the basis for the construction of useful complex features using the construction methods described below. Figure 3C illustrates this for a constructed feature *F*(*i*): “*i* is at most two edges away from a node with degree ≥5.”

Similar to node attributes, also *non unary relations* of R′ can serve as initial features (“rel” in Table 2). Most logic-based or SRL frameworks will allow binary initial features, enabling, e.g., the construction of features like *F*(*i, j, k*) = *edge*(*i, j*)∧*edge*(*i, k*)∧*edge*(*j, k*) expressing that *i, j, k* form a triangle. Such features are outside the reach of most GNN frameworks, though higher-order GNNs (Morris et al., 2019) overcome this limitation.

A special approach that has been proposed in connection with GNNs is the use of *random node attributes* (Abboud et al., 2021; Sato et al., 2021) as initial features. Such random attributes can serve as a substitute for unique node identifiers. Due to their random nature, however, it does not make sense to construct features based on their absolute value, like the one illustrated in Figure 3A. However, they enable the construction of features based on equalities *rid*(*i*) = *rid*(*j*) (*rid* being the random attribute), which with high probability just encodes identity *i* = *j*, and which is robust with regard to the random actual values. One can then construct features like *F*(*i*): “*i* lies on a cycle of length 5” as “*j* is reachable from *i* in 5 steps, and *rid*(*i*) = *rid*(*j*).” A similar capability for constructing equality-based features is presented by Vignac et al. (2020). Here the initial features are in fact unique identifiers, but the subsequent constructions are limited in such a way that again absolute values are not used in an informative manner, and invariance under isomorphisms is ensured.

#### 4.2.2. Construction by aggregation

Complex features are constructed out of simpler ones using simple operations such as Boolean or arithmetic operators, and linear or non-linear transformations. The essential tool for feature construction in graphs, however, is the aggregation of feature values from related entities. The general structure of such a construction can be described as


(9) $$F_{new}(i):=agg\left\{\left|F_{old}(i,j)|j:\phi (i,j)\right|\right\},$$

where *F*_*old*_(**i**, **j**) is an already constructed feature, the delimiters |}, {| are used to denote a multiset, ϕ(**i**, **j**) expresses a relationship between **i** and **j**, and *agg* is an aggregation function that maps the multiset of values *F*_*old*_(**i**, **j**) to a single number. For better readability we here omit the common dependence of *F*_*old*_ and *F*_*new*_ on the input graph (*V*, **R**′). As usual, vector-valued features are also covered by considering each vector component as a scalar feature. In the case of GNNs, Equation (9) takes the special form *F*_*new*_(*i*): = *agg*{|*F*_*old*_(*j*)|*j*:*edge*(*i, j*)|}, and is often referred to as a *message passing* operation. Another special form of aggregation used in GNNs is the *readout* operation that aggregates node features into a single graph level feature: *F*_*readout*_(): = *agg*{|*F*_*node*_(*j*)|*j*:*true*(*j*)|}, where *true*(*j*) stands for a tautological condition that holds for all nodes *j*. D-SRL and I-SRL frameworks are particularly flexible with regard to aggregate feature construction by supporting rich classes of relationships ϕ. U-SRL frameworks, on the other hand, do not support the nested construction of features by (9) in general, and are limited to a single sum-aggregation of basic features *F*_*k*_ via (8).

Applicable aggregation functions *agg* depend on the type of the feature values. When in a logic-based framework all features are Boolean, then *agg* usually is existential or universal quantification. When in a probabilistic framework all feature values are (conditional) probabilities, then *noisy-or* as the probabilistic counterpart of existential quantification is a common aggregator. When features have unconstrained numeric values, then standard aggregators are *sum, mean, min*, or *max*. Table 2 lists under “Aggregation” characteristic forms of aggregation functions in different frameworks (n/a for frameworks that do not support nested aggregations).

In (9), we have written the aggregation function as operating on a multiset. In this form one immediately obtains that if *F*_*old*_ is invariant under isomorphisms, then so is *F*_*new*_. However, in practice the multiset {|*F*_*old*_(**i**, **j**)|…)|} will be stored as a vector. When *agg* then is defined for vector inputs, one has to require that *agg* is *permutation invariant* (which just means that only the multiset content of the vector affects the computed value) in order to ensure invariance under isomorphisms.

#### 4.2.3. Final features

The inductive feature construction described in the preceding subsections leads to features that have a certain *depth* of nestings of aggregation functions. Often this depth corresponds to a maximal radius of the graph neighborhood of **i** that can affect the value *F*(**i**). This example is the case for features constructed by message passing aggregation in GNNs (but not for *readout* features), and the features of the WLK. Many modeling frameworks only are based on features of a limited depth that are obtained by a fixed sequence of feature constructions. This case is denoted by “deep” in the “Final” column of Table 2, whereas “shallow” stands for feature constructions that do not support nested aggregation.

In contrast, I-SRL and R-GNN models are based on an a-priori unbounded sequence of feature constructions that for each input graph (*V*, **R**′) proceeds until a saturation point is reached (“sat” in Table 2). This enables models which are based on final features that are outside the reach of any fixed depth construction. An example is the “contains cycle” graph feature of Example 1.3.

#### 4.2.4. Numeric and symbolic representations

In the previous sections we have considered features mostly at an abstract semantic level. For any given semantic feature there can be very different forms of formal representation. We here illustrate different paradigms on a concrete example. Assume that we have a signature containing a single binary *edge* relation, *l* different Boolean node attributes *a*_1_, …, *a*_*l*_, and a node class attribute *class*. A node classification model may depend on the node feature *F*(*i*) defined as the number of distinct paths of length 2 that lead from *i* to a node *j* for which *a*_1_(*j*) is true. The concrete classification model then can be defined as a logistic regression model based on *F*:


(10) $$P_{\left(V,R\right)}(class(i)=1)=\sigma (aF(i)+b),$$

where *a, b*∈ℝ, and σ denotes the sigmoid function.

In a MP-GNN framework, the construction of *F* and the classification model can be implemented in a two layer network with a generic structure such as


(11) $$\begin{array}{ll} h^{\left(1\right)}(i)= & relu(sum\left\{U^{\left(1\right)}a(h)|h:edge(i,h)|\right\}|⊕V^{\left(1\right)}a(i))\\ h^{\left(2\right)}(i)= & relu(sum\left\{\left|U^{\left(2\right)}h^{\left(1\right)}(h)|h:edge(i,h)\right|\right\}⊕V^{\left(2\right)}h^{\left(1\right)}(i))\\ out(i)= & softmax(Wh^{\left(2\right)}(i)+b) \end{array}$$

where **h**^(*k*)^ are the embedding vectors computed at hidden layer *k*, **a**(*i*) is the attribute vector of *i*, the *U*^(*k*)^, *V*^(*k*)^ and *W* are weight matrices, **b** is a bias vector, ⊕ denotes vector concatenation, and *relu* is component-wise application of the *relu* activation function.

Finally, ***out*** is a two-dimensional output vector whose components represent the probabilities for *class*(*i*) = 1 and *class*(*i*) = 0. The embedding vector **h**^(2)^(*i*) defines a whole set of (scalar) features. The feature *F*(*i*) can be obtained as the first component of **h**^(2)^(*i*) when in both matrices *U*^(1)^ and *U*^(2)^ the first row is set to (1, 0, …, 0). With a suitable setting of *W* and **b**, ***out***(*i*) can then represent the model (10). Clearly, the representational capacity of (11) is not nearly exhausted when used in this manner to implement (10). The architecture (11) would usually encode a model where the output probabilities are a complex function of a multitude of different features encoded in the hidden embedding vectors.

A symbolic representation in a D-SRL framework would take a form like


(12) $$\begin{array}{l} \begin{array}{ccc} \#a_{1}\text{\_}\mathrm{neighbors}\left(i\right) & \leftarrow & \mathrm{sum}\left\{\left|a_{1}\left(h\right)|h:\mathrm{edge}\left(i,h\right)\right|\right\}\\ F\left(i\right) & \leftarrow & \mathrm{sum}\left\{\left|\#a_{1}\_\mathrm{neighbors}\left(h\right)|h:\mathrm{edge}\left(i,h\right)\right|\right\}\\ \mathrm{class}\left(i\right) & \leftarrow & \sigma \left(aF\left(i\right)+b\right) \end{array} \end{array}$$

The first two lines define the feature *F*, while the last implements the classification rule. In contrast to (11), where *F* is defined numerically through the entries in the parameter matrices, it is here defined symbolically in a formal language that combines elements of logic and functional programming languages. The only numeric parameters in the specification are the coefficients *a, b* of the logistic regression model. The representation (12) is more interpretable than (11). However, a new classification model depending on other features than *F* would here require a whole new specification, whereas in (11) this is accomplished simply by a different parameter setting. Also, the symbolic representation will grow in size (and loose in interpretability), when more complex models depending on a large number of features are needed.

Though on different sides of the symbolic/numeric divide, model specifications (11) and (12) are very similar in nature in that they use the same three-step strategy to first define the number of direct *a*_1_-neighbors as an auxiliary node feature, then define *F*, and finally the logistic regression model based on *F*. This iterative construction is not supported in U-SRL frameworks. However, Equation (10) can still be implemented in an U-SRL framework using a specification of the form


(13) $$\begin{array}{l} \begin{array}{cc} \mathrm{class}\left(i\right)\wedge \mathrm{edge}\left(i,j\right)\wedge \mathrm{edge}\left(j,h\right)\wedge a_{1}\left(h\right) & w_{1}\\ \neg \mathrm{class}\left(i\right)\wedge \mathrm{edge}\left(i,j\right)\wedge \mathrm{edge}\left(j,h\right)\wedge a_{1}\left(h\right) & w_{2}\\ \mathrm{class}\left(i\right) & w_{3} \end{array} \end{array}$$

This is an MLN type specification that defines a model of the form (8) with *K* = 3 features defined by Boolean properties and associated weights. The feature *F*_*k*_(**i**) evaluates to *w*_*k*_ if the Boolean property is satisfied by **i**, and otherwise evaluates to zero [**i** = (*i, j, h*) for the first two features, and **i** = (*i*) for the third]. This defines a fully generative model without a separation into feature construction and classification model. However, with suitable setting of the weights *w*_*k*_, the conditional distributions of the *class*(*i*) atoms given full instantiations of the *edge* relation and the node attributes, the model (10) can be obtained.

#### 4.2.5. Expressivity

The general question of expressivity of feature construction frameworks can be viewed from an *absolute* or *comparative* perspective. From the absolute point of view, one asks whether a feature construction framework is generally able to distinguish different entities, i.e., whether for **i**∈(*V*, **R**′) and $\tilde{i}\in \left(Ṽ,{\tilde{R}}^{\prime }\right)$ a feature *F* can be constructed with $F\left(\tilde{i}\right)\left(Ṽ,{\tilde{R}}^{\prime }\right)\neq F\left(i\right)\left(V,R^{\prime }\right)$ (usually only required or desired when **i** and $\tilde{i}$ are not isomorphic). In the context of graph kernels this question was first investigated by Gärtner et al. (2003), who showed that graph kernels that are maximally expressive in this sense will be computationally intractable due to their implicit ability to solve the subgraph isomorphism problem.

**Figure 4:**
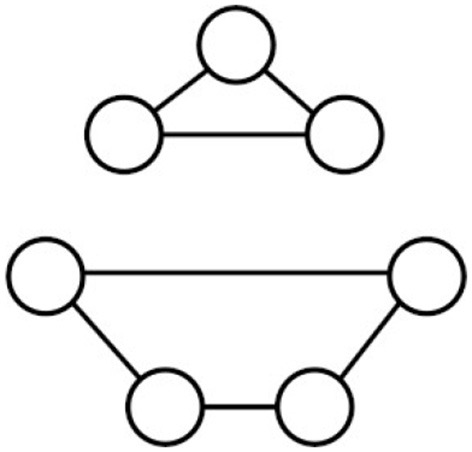
Indistinguishable nodes.

Figure 4 (adapted from Abboud et al., 2021) gives an example of a graph whose nodes do not have any attributes. The nodes on the three-node cycle are not isomorphic to the nodes on the 4-node cycle. However, features that are constructed starting with the vacuous initial feature using the aggregation mechanisms of WLK or GNNs will not be able to distinguish these nodes. As already mentioned in Section 4.2.1, GNNs with random node attributes as initial features, on the other hand, will be able to distinguish the nodes on the three-cycle from the nodes on the four-cycle. Without the need for random node attributes this distinction also is enabled by most SRL frameworks due to their support for binary feature constructions, which here can be used to first construct Boolean features *k-path*(*i, j*) representing whether there exists a path of length *k* from *i* to *j*, and then distinguish the nodes on the three-cycle by the feature *3-cycle*(*i*): = *3-path*(*i, i*).

The discriminative capabilities of feature functions largely depend on the discriminative capabilities of the aggregators (9). It has been suggested that in combination with suitable feature transformation functions applied to *F*_*old*_ before aggregation, and *F*_*new*_ after aggregation, *sum* is a universally expressive aggregation function (Zaheer et al., 2017). As pointed out by Wagstaff et al. (2019), however, the requirements on the transformation functions are not realistic for actual implementations. See also Jaeger (2022) for a detailed discussion.

In the comparative view, one asks whether all features that can be constructed in a framework ABC can also be constructed in a framework XYZ. If that is the case, we write ABC≼_*F*_ XYZ. We use the subscript *F* here in order to emphasize that this is an expressivity relationship about the feature construction capabilities of the frameworks. Different frameworks use features in a different ways, and therefore ABC≼_*F*_XYZ does not directly imply that every model of ABC also can be represented as a model in XYZ. However, feature expressivity is the most fundamental ingredient for modeling capacity.

**Figure 5:**
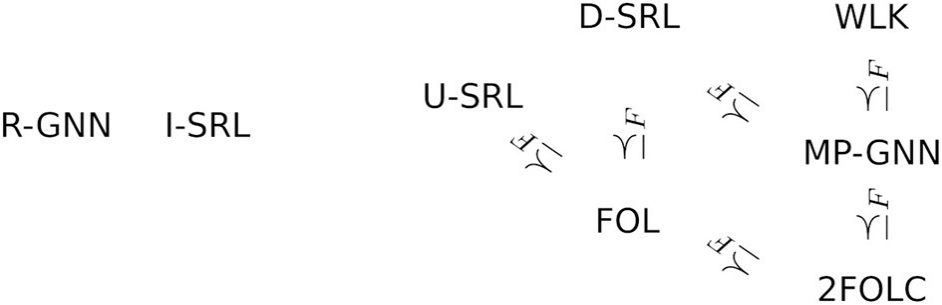
Some expressivity relations.

Figure 5 gives a small and simplistic overview of some expressivity relationships between different frameworks. In this overview we gloss over many technical details regarding the exact representatives of larger classes such as MP-GNN for which the relations have been proven, and the fact that in some cases the comparison so far only has been conducted for graphs with a single edge relation (though generalizations to multi-relational graphs seem mostly straightforward). The relationship FOL≼_*F*_D-SRL has been shown in Jaeger (1997). That MP-GNNs are at least as expressive as the 2-variable fragment of first-order logic with counting quantifiers (2FOLC) is shown by Barceló et al. (2020) on the basis of MP-GNNs that allow feature constructions using both message-passing and readout aggregations. The relationship between MP-GNNs and the Weisfeiler-Lehman graph-isomorphism test was demonstrated by Morris et al. (2019) and Xu et al. (2019). A detailed account of expressivity of different GNN frameworks and their relationship to Weisfeiler-Lehman tests is given by Sato (2020). The MP-GNN≼_*F*_D-SRL relation is demonstrated in Jaeger (2022), and FOL≼_*F*_U-SRL is shown by Richardson and Domingos (2006). It must be emphasized, however, that these results only pertain to the feature expressivity of the frameworks. The reasoning capabilities of logical deduction in FOL are not provided by the SRL frameworks.

The figure also shows that R-GNN and I-SRL are (presumably) incomparable to the other frameworks here shown: their saturation based feature construction, on the one hand, provides expressivity not available to the fixed depth features of the other frameworks. On the other hand, I-SRL has limitations regarding expressing logical features involving negation or universal quantification, and R-GNN cannot aggregate features over a fixed number of steps using different aggregators at each step.

## 5. Reasoning: tasks and techniques

Given a model in the sense of Definition 3.1, we consider several classes of reasoning tasks. Narrowing down the range of possible reasoning tasks considered in Section 1, we are now only concerned with algorithmic reasoning.

### 5.1. Sampling

The sampling task consists of generating a random graph (V,R)∈G(V,R) according to the distribution $P_{\left(V,\tilde{R}\right)}$. Here we consider this sampling problem as a task in itself, which is to be distinguished from sampling as a method for approximately solving other tasks (see below). Sampling as a main reasoning task is mostly considered in the context of graph evolution models, where the comparison of observed statistics in the random sample with real-world graphs is used to validate the evolution model (e.g., Leskovec et al., 2007). A concrete application of sampling from generative models is to create realistic benchmark datasets for other computational tasks on graphs. Bonifati et al. (2020) give a comprehensive overview of graph generator models in this application context.

Sampling is typically a task for fully generative models. However, not all models that are fully generative in the semantic sense of Section 3 necessarily support efficient sampling procedures. This is the case, in particular, for U-SRL models whose specification (8) does not translate into an operational sampling procedure. D-SRL models following the factorization strategy of (6) and (7), on the other hand, allow for an efficient sampling procedure by successively sampling truth values for ground atoms *R*_*k*_(**i**). The same is true for dedicated graph evolution models, and, with slight modifications, for I-SRL models.

While mostly designed for prediction tasks, MP-GNNs have also been adapted as models for random graph generation (e.g., Li et al., 2018; Simonovsky and Komodakis, 2018; You et al., 2018; Dai et al., 2020). Differently from more traditional graph evolution models, which typically only have a small number of calibration parameters, generative GNNs are highly parameterized and can be fitted to training data sets of graphs with different structural properties.

### 5.2. Domain-specific queries

A large class of reasoning tasks falls into the following category of performing inference based on a given model for a single graph under consideration. This covers what in the introduction informally was referred to as reasoning about a single graph, and reasoning about one graph at a time.

Definition 5.1. Let M=(I,μ) be a model. A *domain specific query* is given by

(a.) an input graph $\left(V,\tilde{R}\right)\in I$,
(b.) a *query set* $\rho \subseteq Int_{\left(V,\tilde{R}\right)}(ℛ),$
(c.) a *query objective* that consists of calculating a property of the set $\left\{P_{\left(V,\tilde{R}\right)}\left(R\right)|R\in \rho \right\}$.

Query sets are typically specified by one or several query atoms *r*_*k*_1__(**i**_1_), …, *r*_*k*_*q*__(**i**_*q*_), where ρ then consists of all interpretations **R** in which the query atoms are true. A slightly more general class of queries is obtained when the query atoms can also be negated, with the corresponding condition that the negated atoms are false in **R**. Part (c.) of Definition 5.1 is formulated quite loosely. The most common query objective is to calculate the probability of ρ:


(14) $$P_{\left(V,\tilde{R}\right)}(\rho )=\sum_{R\in \rho }P_{\left(V,\tilde{R}\right)}(R).$$

When ρ is defined by a list of query atoms we can write for $P_{\left(V,\tilde{R}\right)}\left(\rho \right)$ also more intuitively


(15) $$P_{\left(V,\tilde{R}\right)}(r_{k_{1}}(i_{1}),\ldots ,r_{k_{q}}(i_{q})).$$

The prediction tasks of Examples 1.1–1.4 all fall into the category of computing (15) for a single query atom. More general *probabilistic inference* is concerned with computing (15) for general lists of (possibly negated) query atoms. Furthermore, we often are interested in conditional queries of the form


(16) $$P_{\left(V,\tilde{R}\right)}(r_{k_{1}}(i_{1}),\ldots ,r_{k_{q}}(i_{q})|r_{k_{q+1}}(i_{q+1}),\ldots ,r_{k_{p}}(i_{p}))$$

where *r*_*k*_*q*+1__(**i**_*q*+1_), …, *r*_*k*_*p*__(**i**_*p*_) represents *observed evidence*, and *r*_*k*_1__(**i**_1_), …, *r*_*k*_*q*__(**i**_*q*_) represents the uncertain *target* of our inference. If the inference framework permits arbitrary unconditional queries (15), then (16) can be computed from the definition of conditional probabilities as the ratio of two unconditional queries. Thus, Equation (15) already covers most cases of probabilistic inference.

In the most probable genotype problem of Example 3.6 the query set is given by a list of atoms (possibly negated) representing observed genotype data, and the query objective is to compute


(17) $$\arg_{R\in \rho }\max P_{\left(V,\tilde{R}\right)}(R).$$

The computational tools and computational complexity for computing a query differ widely according to the underlying modeling framework, and the class of queries it supports. The simplest case is given by specialized prediction models that only support single (class label) atoms *class*(**i**) as queries. When, moreover, the model (as in GNNs) is directly defined by functional expressions for the conditional probabilities *P*(*class*(**i**)|**R**_*in*_), then the computation consists of a single *forward computation* that is typically linear in the size of the input graph $\left(V,\tilde{R}\right)=\left(V,R_{\text{in}}\right)$ and the model specification. Similarly, performing prediction by a kernel-SVM is usually computationally tractable, though the necessary evaluations of the kernel function here add another source of complexity.

Dedicated classification models provide for efficient predictive inference, because for their limited class of supported queries (14) can be evaluated without explicitly performing the summation over **R**∈ρ. The situation is very different for SRL models that are designed to support a much richer class of queries defined by arbitrary lists of atoms in the R_*out*_ relations, and where the summation in (14) cannot be bypassed. A naive execution of the summation over all **R** is usually infeasible, since the number of **R** one needs to sum over is exponential in the number of unobserved atoms, i.e., atoms that are not instantiated in the input $\tilde{R}$, or included among the query atoms. The number of such unobserved atoms, in turn, is typically polynomial in |*V*|. For SRL models, the basic strategy to evaluate (14) is to compile the distribution $P_{\left(V,\tilde{R}\right)}$ and the query ρ into a standard inference problem in a probabilistic graphical model (e.g., Ngo and Haddawy, 1995; Jaeger, 1997; Richardson and Domingos, 2006), or into a *weighted model counting* problem (e.g., Fierens et al., 2011). These compilation targets are *ground* models in the sense that they are expressed in terms of ground atoms *r*(**i**) as basic random variables. While often effective, they still may exhibit a computational complexity that is exponential in |*V*|. The idea of *lifted inference* is to exploit uniformities and symmetries in the model $P_{\left(V,\tilde{R}\right)}$, which may allow us to sum over whole classes of essentially indistinguishable interpretations **R** at once (Poole, 2003; Van den Broeck, 2011). While more scalable than ground inference in some cases, the potential of such lifted techniques is still limited by general intractability results: Jaeger (2000) shows that inference for single atom queries is exponential in |*V*| in the worst case,^2^ if the modeling framework is expressive enough to support FOL features. Van den Broeck and Davis (2012) obtain a related result under weaker assumptions on the expressiveness of the modeling framework, but for more complex queries.

When exact computation of (14) becomes infeasible, one typically resorts to approximate inference via sampling random **R** according to $P_{\left(V,\tilde{R}\right)}$, and taking the empirical frequency of samples that belong to ρ as an estimate of $P_{\left(V,\tilde{R}\right)}\left(\rho \right)$. When directly sampling from $P_{\left(V,\tilde{R}\right)}$ is not supported (cf. Section 5.1), then this is performed by a *Markov Chain Monte Carlo* approach where samples follow the distribution $P_{\left(V,\tilde{R}\right)}$ only in the limit of the sampling process.

### 5.3. Cross-domain queries

The reasoning tasks considered in the previous section arise when a single known domain of entities is the subject of inference. Cross-domain queries in the sense of the following definition capture reasoning tasks that arise when the exact domain is unknown, or one wants to reason about a range of possible domains at once.

Definition 5.2. Let M=(I,μ) be a model. A *cross-domain query* is given by

- a set J⊆I of input graphs
- a *query set* $\rho =\left\{\rho_{\left(V,\tilde{R}\right)}|\left(V,\tilde{R}\right)\in J\right\}$, where each $\rho_{\left(V,\tilde{R}\right)}\subseteq \text{Int}\left(V,\tilde{R}\right)$
- a *query objective* that consists of calculating a property of the set of sets

(18) $$\{\{P_{\left(V,\tilde{R}\right)}(R)|\;R\in \rho \}|(V,\tilde{R})\in J\}.$$

Definition 5.1 now is just the special case |J| = 1 of Definition 5.2.

Example 5.3. (Deduction, cont.) Consider a logic knowledge base *KB* as a discriminative model as described in Example 3.7. The question whether *KB* implies a statement ϕ then is a cross-domain query where J contains the set of graphs G∈G(<∞,R) in which ϕ does not hold, ρ_*G*_ is the extension of *G* in which *l*_*KB*_ = 1, and the query objective is to decide whether *P*_*G*_(ρ_*G*_) = 0 for all *G*∈J.

Example 5.4. (Model explanation) Consider a GNN model for the graph classification task described in Example 1.3. This will be a discriminative model that takes fully specified graphs representing molecules as input, and returns a probability distribution over the *mutagenic* graph label as output. An approach to *explain* such a model is to identify for a given target value of the graph label, e.g., *mutagenic* = *true*, the input molecule that leads to the highest probability for that label (which can be interpreted as an ideal prototype for the label—according to the model we are trying to explain; Yuan et al., 2020). Finding such an explanation is a cross-domain query in our sense: the set J is the set of all possible input molecules (or the set of all molecules with a given size). For all (*V*, **R**)∈J the query set is the single labeled molecule ρ_(*V*, **R**)_ = {(*V*, **R**, *mutagenic* = *true*)}, and the objective is to find the argmax of (18). This is very similar to the most probable genotype queries considered above, but subtly different: since the underlying model here only is discriminative for the class label, the maximization of (18) does not depend on any prior probabilities for the input graphs (*V*, **R**). Also, what is a cross-domain query for a discriminative model can just be a single domain query for a fully generative model: if here we are looking for an explanation of a fixed size *n*, then we only need to consider the single input domain *V* = [*n*], the query set ρ_(*V*)_ = {([*n*], **R**, *mutagenic* = *true*)|**R**∈*Int*([*n*])(R)}, and the query objective (17). While on the one hand merely a technical semantic distinction, this can make a significant difference when in the first case the existing framework does not directly support the argmax computation for (18), whereas in the second case the computation of (17) may be supported by native algorithmic tools in the framework.

Example 5.5. (Limit behavior, cont.) For a fully generative model consider J=ℐ=G(<∞,∅). For each input graph *G*_*n*_: = ([*n*], ∅) let ρ_*n*_: = ρ_*G*_*n*__ contain the set of graphs with some property of interest, such as being connected, or satisfying a given logic formula. An important cross-domain reasoning task then is characterized by the query objective to determine the existence and value of the limit $\underset{n\to \infty }{\lim}\left[P_{n}\left(\rho_{n}\right)\right]$.

Automated theorem provers provide algorithmic solutions for the reasoning tasks described in Example 5.3. For the analysis of limit probabilities as in Example 5.5 there exist a few theoretical results that puts them into the reach of general automated inference methods (Grandjean, 1983; Jaeger, 1998; Cozman and Mauá, 2019; Koponen and Weitkämper, 2023). However, these results have not yet been carried over to operational implementations.

## 6. Learning: settings and techniques

So far our discussion has largely focused on modeling and reasoning. Our formal definitions in Sections 3 and 5 draw a close link between types of models and the reasoning tasks they support (discriminative, generative, transductive, inductive, …). Turning now to the question of how a model is learned from data, a similar close linkage arises between model types and learning scenarios. Based on the unifying probabilistic view of models according to Definition 3.1, different learning scenarios are essentially just distinguished by the structure of the training data, but unified by a common maximum likelihood learning principle.

The class of input structures I of a model I = (I, μ) characterizes its basic functionality and will be assumed to be fixed a-priori by the learning/reasoning task at hand. What is to be learned is the mapping μ. For this one needs training data of the following form.

Definition 6.1. Let $ℐ\subseteq \tilde{G}(<\infty ,ℛ)$ be given. A dataset for learning the mapping μ consists of a set of examples


(19) $$(V_{n},{\tilde{R}}_{n}),{\tilde{R}}_{n}^{+}\;(n=1,\ldots ,N),$$

where $(V_{n},{\tilde{R}}_{n})\in ℐ$, and ${\tilde{R}}_{n}^{+}\in \tilde{G}(V_{n},ℛ)$ complements ${\tilde{R}}_{n}$ in the sense that ${\tilde{R}}_{n,i}^{+}\left(i\right)\neq ?\Rightarrow {\tilde{R}}_{n,i}\left(i\right)=?$ (*i* = 1, …, *m*).

The unifying learning principle is to find the mapping μ that maximizes the log-likelihood


(20) $$\sum_{n=1}^{N}\log\mu (V_{n},{\tilde{R}}_{n})({\tilde{R}}_{n}^{+}).$$

In practice, the pure likelihood (20) will often be modified by regularization terms or prior probabilities, which, to simplify matters, we do not consider here. Furthermore, some learning objectives, such as the max-margin objective in learning a kernel-SVM, are not based on the log-likelihood as the central element at all. However, as the following examples show, Equation (20) still covers a fairly wide range of learning approaches.

Example 6.2. (Node classification with GNN) Consider R consisting of a single binary *edge* relation, node attributes *a*_1_, …, *a*_*k*_, and a node label *l*. For training a discriminative model with R_*in*_ = {*edge, a*_1_, …, *a*_*k*_}, and R_*out*_ = {*l*}, the training examples consist of complete input graphs (*V*_*n*_, **R**_*n*_)∈G(< ∞, R_*in*_), and ${\tilde{R}}_{n}^{+}$ is a partial interpretation ${\tilde{L}}_{n}$ of *l*. In the transductive setting, *N* = 1, and (*V*_1_, **R**_1_) is equal to the single input graph for which the model is defined. Under the factorization (7) the log likelihood (20) then becomes


$$\sum_{n=1}^{N}\sum_{i:{\tilde{L}}_{n}(i)\neq ?}\log\mu (V_{n},R_{n})({\tilde{L}}_{n}(i)).$$

The usual training objective for GNNs of minimizing the *log-loss* is equivalent to maximizing this log-likelihood.

Example 6.3. (Generative models from incomplete data) The discriminative learning scenario of Example 6.2 requires training data in which input relations R_*in*_ are completely observed. When also some of the attributes *a*_*i*_, and maybe the *edge* relation are only incompletely observed in the training data, then no valid input structure for a discriminative model is given. However, no matter which relations are fully or partially observed, a partial interpretation ${\tilde{R}}_{n}$ can always be written as a valid training example


(21) $$(V_{n},\emptyset ),{\tilde{R}}_{n}$$

for a fully generative model. Without any assumptions on the factorization of the distributions *P*_*n*_, the log-likelihood now takes the general form


$$\sum_{n=1}^{N}\log\mu (V_{n},\emptyset )({\tilde{R}}_{n})=\sum_{n=1}^{N}\;\;\sum_{R\in Int_{\left(V_{n},{\tilde{R}}_{n}\right)}ℛ}\log\mu (V_{n},\emptyset )(R).$$

While always well-defined, this likelihood may be intractable for optimization. An explicit summation over all $R\in \text{Int}\left(V_{n},R_{n}\right)\left(R\right)$ is almost always infeasible. When optimization of the *complete data likelihood* μ(*V*_*n*_, ∅)(**R**) for R∈IntV(R) is tractable, then the *expectation-maximization* strategy can be applied, where one iteratively imputes expected completions **R**_*n*_ for the incomplete observations **R**_*n*_ under a current model μ, and then optimizes μ under the likelihood induced by this complete data. For U-SRL models, even the complete data likelihood usually is intractable due to its dependence on the *partition function* *Z* = *Z*(μ) in (8). In this case the true likelihood may be approximated by a *pseudo-likelihood* (Besag, 1975; Richardson and Domingos, 2006).

Example 6.4. (Learning logic theories) Consider now the case of a logical framework where a model is a knowledge base *KB* that we consider as a discriminative model in the sense of Example 3.7. Learning logical theories or concepts is usually framed in terms of learning from positive and negative examples, where the example data can consist of full interpretations, logical statements, or even proofs (De Raedt, 1997). The learning from interpretations setting fits most closely our general data and learning setup: examples then are fully observed graphs *G*_*n*_ = (*V*_*n*_, **R**_*n*_) together with a label ${\tilde{R}}_{n}^{+}=L_{n}\in \left\{0,1\right\}$, and optimizing (20) amounts to finding a knowledge base *KB* such that *KB* is true in all *G*_*n*_ with *L*_*n*_ = 1, and false for *G*_*n*_ with *L*_*n*_ = 0, i.e., the standard objective in logic concept learning.

### 6.1. Numeric and symbolic optimization

Examples 6.22–6.4 present a uniform perspective on learning in very different frameworks. However, the required techniques for solving the likelihood optimization problem are quite diverse. Corresponding to the combination of symbolic and numeric representation elements of a modeling framework, the learning problem decomposes into a *structure learning* part for the symbolic representation, and a *parameter learning* part for the numeric elements. Since the numeric parameterization usually depends on the chosen structure, this can lead to a nested optimization in which structure learning is performed in an outer loop that contains parameter learning as an inner loop. Structure learning amounts to search in a potentially infinite combinatorial space. Parameter learning, on the other hand, typically is reduced to the optimization of a differentiable objective function, for which powerful gradient-based methods are available.

The (empirical) fact that numeric optimization of parameters is somewhat easier than combinatorial optimization of symbolic structure favors frameworks that are primarily numeric, notably GNNs. As illustrated in Section 4.2.4, feature constructions that in other frameworks require symbolic specifications (12), (13) here are encoded in numeric parameter matrices (11). As a result, learning that in symbolic representations requires a search over symbolic representations, here is accomplished by numeric optimization. However, even GNNs are not completely devoid of “symbolic” representation elements: the neural network architecture is a component of the model specification in a discrete design space, and finding the best architecture via *neural architecture search* (Elsken et al., 2019) leads to optimization problems in discrete, combinatorial search spaces that have much in common with structure learning in more manifestly symbolic frameworks.

While presenting a harder optimization task for machine learning, symbolic, structural parts of a model may also be supplied manually by domain experts, thus reducing the machine learning task to the optimization of the numeric parameters. Many SRL frameworks, so far, rely to a greater or lesser extent on such a humans-in-the-loop scenario.

## 7. Integration

In view of the complementary benefits of symbolic and numeric models, it is natural to aim for combinations of both elements that optimally exploit the strengths of each. Emphasizing neural network frameworks as the prime representatives for numeric approaches, these efforts are currently mostly pursued under the name of *neuro-symbolic integration* (Sarker et al., 2021). An important example in the context of this review is the integration of the I-SRL ProbLog framework with deep neural networks (not specifically GNNs). The underlying philosophy for the proposed *DeepProbLog* framework (Manhaeve et al., 2018) is that neural frameworks excel at solving low-level “perceptual” reasoning tasks, whereas symbolic frameworks support higher-level reasoning. The integration therefore consists of two layers, where the lower (neural) layer provides inputs to the higher (symbolic) layer.

The unifying perspective on model semantics and model structure we have developed in Sections 3 and 4 gives rise to more homogeneous integration perspectives: a conditionally generative model that is constructed via the factorization (6), (7) consists of individual discriminative models (7) for each relation *R*_*k*_ as building blocks. In principle, different such building blocks can be constructed in different frameworks, and combined into a single model via (6). Moreover, this approach will be consistent in the sense that if all constructions of the component discriminative models use (20) as the objective, then the combination of these objectives is equivalent to the maximizing the overall log-likelihood μ(*V*_*n*_, **R**_*in*_)(**R**_*out*_) directly for the resulting conditional generative model. Here we deliberately speak of “constructing” rather than “learning” component models in order to emphasize the possibility that some model components may be built by manual design, whereas others can be learned from data.

Piecing together a combined model from heterogeneous model components only is useful when the resulting model then can be used to perform inference tasks. This will limit the reasoning capabilities to tasks that can be broken down into a combination of tasks supported by the component models. A possible alternative is to compile all individual components into a representation in a common framework with high expressivity and flexible reasoning capabilities. As shown in Jaeger (2022), quite general GNN architectures for representing discriminative models can be compiled into an RBN representation, and integrated as components into a bigger generative model. While theoretically sound, it is still an open question whether this approach is practically feasible for GNN models of the size needed to obtain high accuracy on their specialized discriminative tasks.

## 8. Conclusion

We have given a broad overview over modeling, reasoning, and learning with graphs. The main objective of this review was to view the area from a broader perspective than more specialized existing surveys, while at the same time developing a coherent conceptual framework that emphasizes the commonalities between very diverse approaches to dealing with graph data. Our central definitions of models, reasoning types and learning tasks cover a wide range of different frameworks and approaches. While the uniformity we thereby obtain sometimes is a bit contrived (notably by casting logical concepts in probabilistic terms), it still may be useful to elucidate the common ground among quite disparate traditions and approaches, to provide a basis for further theoretical (comparative) analyses, and to facilitate the combination and integration of different frameworks.

## Author contributions

The author confirms being the sole contributor of this work and has approved it for publication.

## Footnotes

^1^In logic terminology, these graphs would be called the models of *KB*. This is in direct conflict with our terminology, where rather the knowledge base is considered a model; therefore we avoid to use the term “model” in the sense of logic.

^2^Subject to the complexity-theoretic assumption that NETIME≠ETIME.
